# The Evolving Phenotypes of Cardiovascular Disease during COVID-19 Pandemic

**DOI:** 10.1007/s10557-021-07217-8

**Published:** 2021-07-30

**Authors:** Michele Correale, Francesca Croella, Alessandra Leopizzi, Pietro Mazzeo, Lucia Tricarico, Adriana Mallardi, Martino Fortunato, Michele Magnesa, Vincenzo Ceci, Alessandra Puteo, Massimo Iacoviello, Matteo Di Biase, Natale Daniele Brunetti

**Affiliations:** 1 Policlinico Riuniti University Hospital, Foggia, Italy; 2 Department of Medical and Surgical Sciences, University of Foggia, Foggia, Italy

**Keywords:** Coronavirus disease 2019 (COVID-19), Severe acute respiratory syndrome coronavirus 2 (SARS-CoV-2), Thrombosis; Heart failure, Acute coronary syndrome, Arrhythmias, Cardiovascular disease

## Abstract

COVID-19 pandemic has negatively impacted the management of patients with acute and chronic cardiovascular disease: acute coronary syndrome patients were often not timely reperfused, heart failure patients not adequately followed up and titrated, atrial arrhythmias not efficaciously treated and became chronic. New phenotypes of cardiovascular patients were more and more frequent during COVID-19 pandemic and are expected to be even more frequent in the next future in the new world shaped by the pandemic. We therefore aimed to briefly summarize the main changes in the phenotype of cardiovascular patients in the COVID-19 era, focusing on new clinical challenges and possible therapeutic options.

## Introduction

### What’s Happening to Patients with Cardiovascular Disease during the Pandemic of COVID-19

During the COVID-19 pandemic, fear and anxiety are two important emotional aspects to consider when analyzing changes in cardiology practice. Media hype, stay-at-home mandates, and fear of in-hospital contagion discouraged too many patients from accessing emergency medical services and ambulatory care, even in case of severe cardiovascular conditions. The transformation of many hospital wards into COVID-19 units and the adoption of protocols against contagion with the deferral of non-urgent hospitalizations and ambulatory access, induced in many patients with complex disease, such those with cardiovascular, metabolic, and rheumatologic disease, a sense of being left to oneself.

COVID-19 pandemic has negatively affected also the management of patients with acute cardiovascular disease (CVD), especially those with acute coronary syndromes (ACS). De Rosa et al. [1] showed in a multicenter, nationwide survey a 48% reduction of AMI admissions during the week 12–19 March 2020, compared to the same period in 2019. The reduction for non-ST elevation myocardial infarction (NSTEMI) admissions was higher than for ST elevation myocardial infarction (STEMI) (65% vs 26%). Piccolo et al. [2] found a 32% decline in the number of coronary angioplasties (PCIs) for ACS with in data from 20 PCI centers comparing the 4 weeks before the outbreak of pandemic in Italy (from January 30 to March 26, 2020) with the following 4 weeks. Moreover, the decline in PCI rates was similar when compared to the same period of 2019 (36–38%). Significant delays from symptom onset to first medical contact and from medical contact to coronary revascularization have been reported in patients with acute myocardial infarction (AMI) [3]. A dramatic reduction of telemedicine access for CVD was observed in March 2020 in Italy compared with March 2019. The reduction was substantially consistent for all electrocardiogram findings, ACS, other acute CVD and normal [4]. This can be presumably explained by the fear of in-hospital contagion, a congestion of the emergency system overwhelmed by COVID-19 patients and an implementation of precautionary measures against contagion, leading to an increase in STEMI case fatality and major complications.

### Main Cardiovascular Complications after COVID-19 Disease

Patients with CVD are more susceptible to COVID-19 and have a more severe clinical course once infected. The cardiac manifestations of COVID-19 include cardiac arrhythmias, myocarditis, pericarditis, ACS, heart failure (HF), cardiogenic shock, and cardiac arrest [5, 6]. All these conditions can precede and occur in absence of pulmonary or other types of complications, long after viral clearance and recovery, and be more associated with mortality than does respiratory disease [7, 8].

In patients with COVID-19 known conduction system or sinus node disease or new-onset high degree AV block or sinus node dysfunction, may occur and exacerbate, mainly in case of myocardial involvement [9]. Other mechanisms of AV block in COVID-19 may be vagally mediated due to neuroinvasion or hypoxia [10]. Toniolo et al. [11] observed, in March 2020, a higher proportion of pacemaker implantations for syncope compared to the same period of 2019.

In a retrospective study, heart palpitations were reported as an initial symptom in 7.3% of patients admitted with diagnosis of COVID-19 [12]. Atrial fibrillation was the most frequent sustained arrhythmia, being reported in 17% of COVID-19 patients during hospitalization. In hospital malignant ventricular arrhythmias (sustained ventricular tachycardia or fibrillation) occurred in 5.9% of COVID-19 patients and were more frequent in patients with elevated troponin levels [13]. New-onset malignant ventricular arrhythmia may be therefore considered a marker of acute myocardial injury.

SARS-CoV-2 may induce the activation of the coagulation cascade, leading sometimes to severe hypercoagulability, platelet activation, endothelial dysfunction, or vaso-constriction, with consequent venous thromboembolism, cor pulmonale, systemic and pulmonary arterial thrombosis and embolism, ischemic stroke, and myocardial infarction (AMI) [14–17].

Another initial presentation of COVID-19 infection is ACS, either as STEMI or NSTEMI/unstable angina [18–20]. Myocardial ischemia and AMI could be secondary to plaque rupture triggered by a virus-induced stress response or from thrombosis secondary to hypercoagulability [21]. Increased heart rate, hypoxia, and hypotension may precipitate an ischemia–supply imbalance in patients with coronary artery disease (CAD), but also potentially in those with cardiac hypertrophy and microvascular dysfunction [22].

In patients with COVID-19 infection, myocardial injury, defined as elevation in cardiac troponin concentration above the 99th percentile of upper reference limit, could be multifactorial and include atherosclerotic plaque rupture, coronary vasospasm, hypoxic injury to the vasculature, direct endothelial, or formation of microthrombi [23]. Myocardial injury and fulminant myocarditis can occur from direct viremic effect on the myocardial cells and secondary effects from the body’s hyperimmune response to the virus and overall systemic inflammatory response without direct viral infiltration [24, 25].

Elevated cytokine levels can also lead to myocardial injury and predispose to atrial and ventricular arrhythmias [26]. Increased levels of inflammatory cytokines and respiratory distress can exacerbate pre-existing left ventricular (LV) dysfunction or lead to a new onset cardiomyopathy. Myocarditis, stress cardiomyopathy, or myocardial ischemia may underlie new onset LV dysfunction. Right ventricular (RV) HF may occur following elevated pulmonary artery pressure after pulmonary complications [27]. In early stages, exacerbation of HF with preserved ejection fraction (HFpEF) can occur after aggressive fluid resuscitation attempts, and, in later stages of the disease, in case of raised cytokine levels, acute systolic HF leading to cardiogenic shock has been reported [25].

Patients with HF are at a higher risk of severe disease and mortality in case of COVID-19 [28]. Symptoms of both conditions may overlap and enhance each other. Even after the negative molecular test for SARS-Cov-2 search, several COVID-19 patients complain of dyspnea for a long time. In older patients with pre-existing CAD, HF may be caused by worsening demand–supply balance, while myocarditis is more likely the cause in younger patients [29].

An impaired blood pressure regulation may occur in critically ill patients, manifesting with either profound hypotension or hypertension; whether this is a reaction to the illness or a sign of potential derangements in ACE2 expression is still unclear [30, 31]. Current data suggests that CVD, cardiac manifestations, and cardiac injury in COVID-19 are clinically relevant predictors of overall disease severity and mortality [32].

## Changing Phenotypes in Cardiovascular Diseases after COVID-19 Pandemic

### Coronary Artery Disease

Stefanini et al. [33] demonstrated that up to 60% of STEMI patients affected by COVID-19 had true-culprit lesion vessel disease, while the remaining part just mimed the STEMI condition. In this latter group of patients, it was very hard to ascertain whether the STEMI-like clinical presentation was caused by a type-2 AMI, a myocarditis subsequent to SARS-CoV-2 infection, the SARS-CoV-2-related endothelial dysfunction, or a cytokine storm. COVID-19 patients, either (true) STEMI or not, actually need to be followed up to define the correct diagnosis but also to prevent and early diagnose severe cardiac involvement and damage. During the COVID-19 pandemic, there was a more than threefold increase in mortality from STEMI and a significant increase in out-of-hospital cardiac arrests [34]. The lack of appropriate and timely revascularization for patients with ACS might have other important clinical consequences, not yet measured, including increased risk for HF or sudden cardiac death (SCD) [2]. Cammalleri et al. [35] observed a 63% reduction of patients with STEMI admitted to cath labs, a higher prevalence of patients with TIMI flow ≤ 2 at the end of PCI procedures, and a greater use of glycoprotein IIb/IIa inhibitors from 1 to 31 March 2020, compared to the same period of 2019. Lower LVEF at baseline and at discharge and longer hospital stay were also observed. Given that non-revascularized patients are more likely to develop worse ventricular remodelling after ACS, more severe phenotypes of HF, more symptomatic, and earlier-onset cases of HF are to be expected [36]. Delayed reperfusion increases CAD complications, ventricular arrhythmia, systolic disfunction, cardiac arrest, and higher mortality. CV risk factors and other comorbidities negatively affect clinical outcome [37].

### Heart Failure

Patients with HF are at a higher risk of severe disease and mortality with COVID-19 [28], and the overall management of HF patients was dramatically affected by COVID-19. Symptoms of both conditions may overlap and they may potentiate each other. Furthermore, patients even after the negative test for SARS-Cov-2 complain of dyspnea for a long time [38]. Treating HF patients after SARS-Cov-2 infection may represent a challenging task. A significant decline in hospitalization rates for acute HF during the COVID-19 pandemic is described as a consequence of the fear for infection [39].

The number of HF hospitalizations was significantly reduced during the COVID-19 pandemic, compared to 2019 (p < 0.001); in-hospital mortality was significantly higher in 2020 than in 2019 (p = 0.015) [40].

Remarkably, hospitalized patients had more severe symptoms on admission, possibly suggesting longer decision time before hospital access or missing less severe cases. General and partial lockdown surely limited SARS-CoV-2 contagion, but also medical contacts and care. Regular follow-up visits for HF patients were delayed or deleted, leading to later diagnosis and treatment of episodes of decompensation and missed opportunities for the optimization of medical and nonmedical therapy. This potential undertreatment of HF may impact long-term prognosis [41]. Close monitoring of electrolyte and renal function is critical to safe dose adjustment of guideline-directed therapies for HF with reduced ejection fraction and diuretics [42]. Lifestyle changes during lockdown, such as dietary changes, increased alcohol consumption, and decreased physical activity, may increase episodes of HF decompensations [43, 44]. The postponement and cancellation of elective diagnostic and therapeutic procedures blocked the work-up of patients with advanced HF awaiting a LVAD or heart transplantation (HT) [15, 45], with predictable long-term negative consequences [39]. Patients undergoing LVAD/HT evaluation may experience delays in listing and/or surgery leading to worsening nutritional, functional, or hemodynamic status. As a result, the future incidence, prevalence, and severity of HF are reasonably expected to increase, with expected new phenotype of HF patients [39] (higher percentage of patients refractory to standard HF therapies, with more advanced NYHA functional class, with lower mean values of LVEF and more frequent patterns of advanced diastolic dysfunction and RV systolic dysfunction, uncontrolled comorbidities and higher percentage of non-target ACE-i/ARBs/ARNI dosage patients).

### Arrhythmias

The arrhythmic risk in COVID-19 patients seems to be related to several factors as increased sympathetic activity, systemic inflammatory response, and myocardial injury. These substrates can predispose to both atrial and ventricular arrhythmias [46]. Quarantine limitations may induce a chronic stress condition that activates the sympathetic system and promotes a neuroendocrine dysregulation which might precipitate the occurrence of arrhythmias [47]. Several studies have shown a positive correlation between psychosocial stressors and arrhythmias [48]. The psychological impact of COVID-19 pandemic on patients with arrythmias was significant. Not-urgent procedures were postponed and routine outpatient visits were either cancelled or remotely held [49]. The lower admission rate for atrial fibrillation (53.4%) was observed [1]. A significant increase in sudden cardiac death (SCD) events (58%) was reported in northern Italy, and this was attributed to late complications of AMI or ischemia [43, 50].

The current reduction in CV admissions and routine outpatient activity might be followed by two profoundly different phenomena: either a rebound of patients with cardiovascular conditions requiring intensive treatment, or a more blunted recovery [44]. An increase in arrhythmic phenomena in the next months due to higher stress levels and an unhealthy lifestyle [43] and an increase in hospitalizations for HF decompensation due to AF and tachyarrhythmias not treated in the previous months is expected after COVID-19. The reduced treatment of atrial arrhythmias in recent months [51] could favor a chronicization of these arrhythmias in the next future. New-onset AF in a patient with established HF is associated with a worse outcome, probably because a marker of a worse clinical conditions and directly impairing cardiac function; patients with chronic HF and permanent AF show a worse outcome than those in sinus rhythm [52]. New recommendations electrophysiology and implantable device procedures have been issued by the ESC for the diagnosis and management of CV disease during the COVID-19 pandemic [53]. For monitoring and follow-up of patients with cardiac implantable devices, remote monitoring should be utilized as much as possible; elective ablation and cardiac device implantation procedures should be postponed and urgent procedures should only be performed in exceptional cases after careful consideration of all pharmacological treatment options. In hospitalized patients with AF/atrial flutter without hemodynamic instability, rate control should be favored rather than the use of anti-arrhythmic drugs (AADs) to avoid interactions with antiviral drugs. Routine cardiovascular care suspension (including catheter ablation) would be expected to result in deterioration of chronic cardiovascular conditions, increased admissions, and higher morbidity and mortality.

In the management of arrhythmias, drug-drug interactions including antiviral, antiarrhythmic, and anticoagulation drugs should be considered before administration [54]. Some of the drugs used for the COVID-19 infection, as chloroquine/hydroxychloroquine, azithromycin lopinavir/ritonavir, may have interactions with myocardial cells, especially during the repolarization phase, and may cause a risk of prolonging the QTc interval and torsade de pointes [55].

In critically ill patients with COVID-19 infection and recurrent VTS and VF or hemodynamic instability due to new onset AF/atrial flutter, i.v. amiodarone is the antiarrhythmic medication of choice. However, its combination with hydroxychloroquine and/or azithromycin should be preferably avoided and the benefit of treatment should be balanced against the increased proarrhythmic risk due to QT prolongation [53]. Santoro et al. found that during hospitalization for COVID-19, 14% of patients developed QTc prolongation; dual antiviral therapy, age, and basal heart rate were the only independent predictors of QT prolongation [56]. Intravenous lidocaine may be considered a safer but less effective alternative to amiodarone, especially if underlying myocardial ischemia is suspected [10]. Recent recommendation from the Mayo Clinic is that if there is QTc prolongation > 500 ms or increase in QTc > 60 ms after initiation of COVID-19 treatment, stopping medications or reverting QTc prolonging effect should be considered [57].

### Myocarditis

Systemic inflammatory response such as cytokine storm and direct viral infection of the myocardium can induce fulminant myocarditis, one of the recognized cardiovascular complications of COVID-19, characterized by sudden and severe diffuse inflammation of myocardium, which can often lead to ventricular arrhythmias, cardiogenic shock, and death [13]. Human coronavirus-associated myocarditis is known. One study suggested that up to 7% of COVID-19 related deaths were due to myocarditis [6]. The clinical presentation of SARS-CoV-2 myocarditis varies from mild symptoms, such as fatigue and dyspnea, to chest pain or chest tightness during exertion. Many patients worsen, leading to acute-onset HF with cardiogenic shock. The most emerging presentation is fulminating myocarditis, defined as ventricular dysfunction and HF within 2–3 weeks of contracting the virus [58].

Cardiac magnetic resonance (CMR) represents the non-invasive gold standard technique for myocardial tissue characterization. However, in addition to traditional disadvantages of CMR, new obstacles limit the use of CMR during COVID-19 pandemic, such as assessment of critically ill patients and proper disinfection of scanner and room. Therefore, CMR is frequently not possible in the critically ill patient and has to be held until the patient is clinically stable. This potential diagnostic gap can be filled by CT, as it can be used for describing pulmonary parenchymal involvement, coronary anatomy and pulmonary vasculature, wall motion abnormalities and partly myocardial tissue characterization [59]. The management of myocarditis secondary to COVID-19 should be similar to myocarditis with other etiologies; no specific guidelines are available. However, aggressive supportive measures, including the use of temporary mechanical circulatory support devices, may be necessary [60]. Reorganization of Myocarditis Disease Unit in “Tele-MDU” to transfer multidisciplinary activity on a virtual platform was described [61].

### Pulmonary Hypertension

Acute pulmonary hypertension (PH) was frequently reported in patients affected by severe COVID-19 [62]. Patients with severe COVID-19 showed a higher proportion of PH than mild COVID-19 disease (22% vs 2%). Among hospitalized COVID-19 patients, PH was associated with signs of more severe COVID-19 and with worse in-hospital clinical outcome. Elevated pulmonary artery systolic pressure by echocardiography was predictive of mortality [63]. Thickened pulmonary vascular walls, one important hallmark of pulmonary arterial hypertension, were described in patients died of COVID-19 [64]. COVID-19 patients with chronic obstructive pulmonary disease, congestive HF, myocardial injury, pulmonary embolism, and prior PH were at a higher risk of worsening PH.

Data from a prospective, multicenter, observational study revealed 41% of all subjects with persistent symptoms 100 days after COVID-19 onset, with dyspnea being most frequent (36%). Patients still showed an impaired lung function, with a reduced diffusing capacity in 21% of the cases. Signs of PH were only present in a minority of patients [65]. In COVID-19 infection, PH and RV failure are common complications [66], being reported after the recovery even in moderate cases. PH can occur after a COVID-19 viral infection [67].

PH can develop unusually rapidly following COVID-19 pneumonia and was most likely caused by progressive pulmonary parenchymal abnormalities combined with microvascular damage of the pulmonary arteries [68].

Among patients with severe COVID-19 disease and PH, complications including acute respiratory distress syndrome, acute myocardial injury, intensive care unit admission, mechanical ventilation, and mortality rates are higher [69]. Patients with COVID-19 who develop PH are generally characterized by longer illness, greater reduction in exercise tolerance, and slower recovery of saturation.

### Comorbidities (Renal Failure, Diabetes Mellitus, Hypertension)

**Fig. 1:**
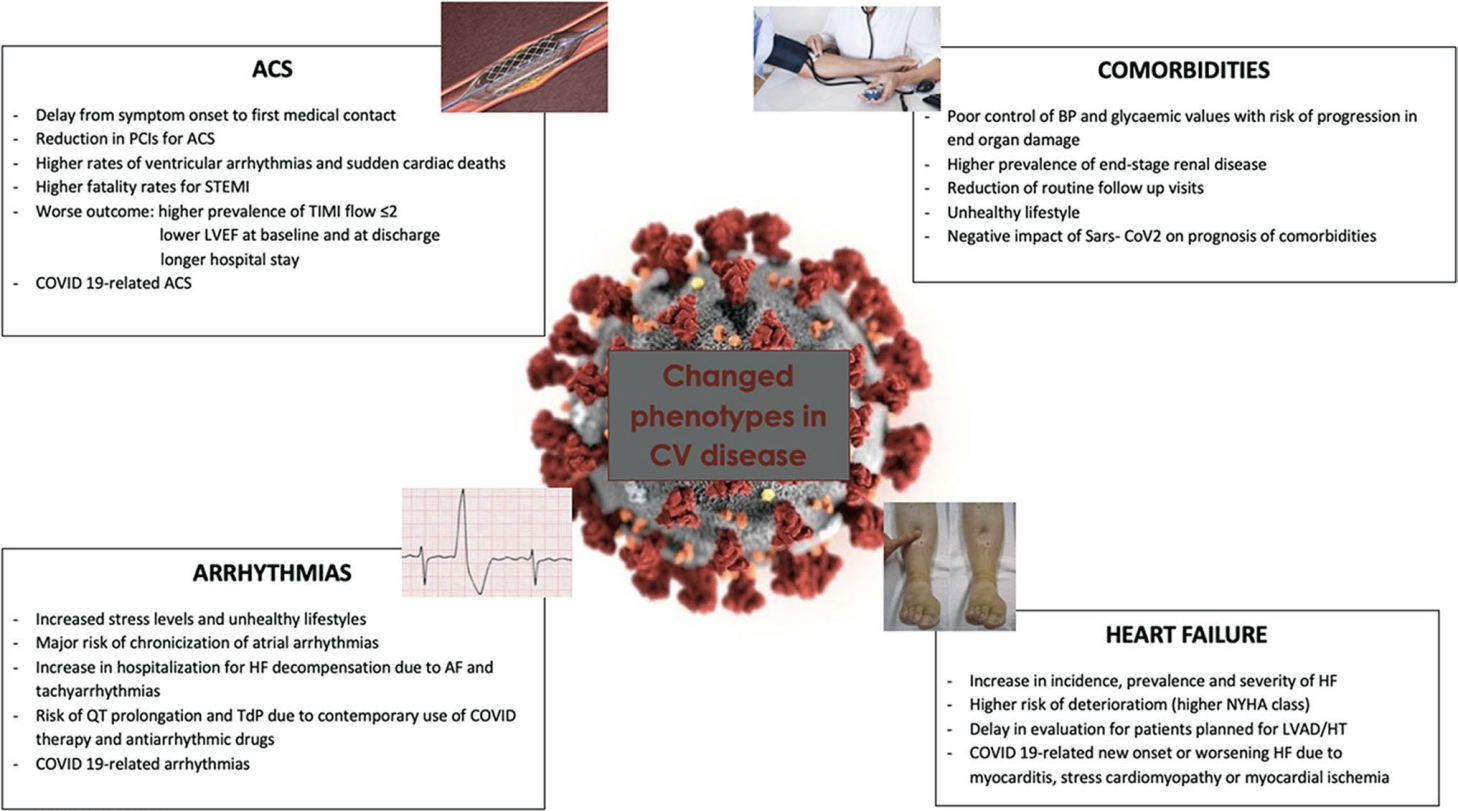
New phenotypes of cardiovascular disease in the COVID-19 era

During COVID-19 pandemic, the management of patients with chronic diseases, such as hypertension, renal failure, and diabetes mellitus (DM), represented a relevant problem [70]. These comorbidities are often found in CAD and HF patients [71], and may modulate the prognosis in case of SARS-Cov-2 infection [53]. New phenotypes (Fig. 1) of CV patients were determined by the reduction of medical assistance, resulting from the pandemic, in case of hypertension, diabetes, or renal failure. Hypertension was associated with an increased composite poor outcome of mortality, severe COVID-19, ARDS, need for ICU care, and disease progression in patients with COVID-19 [72]. One concern in the first phases of epidemic was represented by the possible interaction between angiotensin receptor blockers (ARBs) and ACE with COVID-19 due to the discovery that ACE2 was necessary for viral entry. Optimal management of hypertension can however as usual contribute to a better prognosis in COVID-19 [73]; the Council on Hypertension of the European Society of Cardiology (ESC) strongly recommends that patients should continue treatment with their usual anti-hypertensive therapy because there is no clinical or scientific evidence to suggest that treatment with ACEi or ARBs should be discontinued because of the COVID-19 infection.

In the COVID-19 era, the challenge is to achieve target BP control in a “New Normal” lifestyle, in which health care workers may have a reduced opportunity for in-person clinical examination of patients [73]. The remote blood pressure monitoring system using telemedicine was introduced in achieving target levels [74].

The kidney involvement of the SARS-CoV-2 infection should be associated with three different clinical settings. The first includes patients with chronic kidney disease (frequent comorbidity in CHF and coronary heart disease (CHD)), the second includes patients with acute kidney injury (AKI) (frequently present in AHF), and the third includes immunosuppressed patients such as renal graft recipients or patients with glomerular diseases [75]. Kidneys are a target organ of COVID-19 damage. Visceral tubular and glomerular epithelial cells have been shown to be the primary target cells for SARS-CoV-2 [76]; this makes them susceptible to the cytopathic effects of the virus in the case of viremia [77] and is underlying the high incidence of AKI in severely infected patients [78]. It was argued that renal involvement could be an early sign of severe infection. Proteinuria, hematuria, increased creatinine levels, BUN, and AKI are independent risk factors for hospital death [79]. Renal replacement therapy (RRT) indications for COVID-19-related AKI are not different from any other AKI. Early initiation of RRT can increase the risk of contamination for healthcare professionals and increase the burden for nephrology practice [12]. Some treatments for SARS-Cov-2 infection, such as cortisone therapy, are contraindicated for patients with CHF and for those with chronic kidney disease.

Glucose metabolism disorders are highly prevalent in HF and in CVD patients and impact on disease progression and on long-term morbidity and mortality significantly. The presence of diabetes seems to be independently associated with COVID-19 severity and increased mortality [14–16]. Glycemic deterioration is a typical complication of COVID-19 in patients with impaired glucose regulation or diabetes and in patients requiring insulin, SARS-CoV-2 infection was associated with a rapidly increasing need for high doses of insulin [17]. Patients with diabetes and COVID-19 should be carefully monitored for their adherence to prescribed medications (including insulin injections) and their blood levels of glucose, which should be checked more frequently. Patients with diabetes should carefully maintain a healthy lifestyle and control potential risk factors. Glycemic control during infectious diseases, however, is often suboptimal, and antidiabetic drugs and insulin therapy have to be adapted accordingly. The COVID-19 pandemic is driving significant changes in the healthcare system and disrupting current best practices for diabetic limb preservation, leaving large numbers of patients without adequate care [21]. Some authors support triage systems that help reduce hospital visits for non-fatal wounds, allocating patients with less severe problems to office visits or even telemedical care and remote monitoring [21]. Calling on people to stay at home will most likely reduce the amount of physical exercise compared to usual daily routine. Patients with COVID-19 should furthermore be re-educated in recognition and handling of diabetic ketoacidosis since infection is one of its most frequent triggers [28]. Telemedicine and other innovative strategies could be a reasonable approach to at least partly mitigate the problem of uncontrolled diabetes [30].

### Are Cardiologists Ready to Treat Patients with CV Disease after the Pandemic?

The COVID-19 pandemic has affected not only the world population, but also the health care system in every country and its organizational framework [80]. Major changes in the design of EDs have been proposed for revised triage and surveillance protocols, and new pathways to separate suspected cases; “fever units” have also been implemented in EDs [81, 82]. Only cases that are proved negative are transferred to non-COVID-19 wards, while patients tested positive undergo special care [83]. Many ED worldwide [84] defined different isolation spaces, examined and triaged patients prior to ED admission, and instituted separated area for general or critical ill emergency patients and for patients with suspected COVID-19, fever and respiratory symptoms. Following such revised protocol, the temporary ED closures were drastically reduced after COVID-19 contamination [85].

Staff has to be trained and educated on every aspect of contaminated infectious diseases, including the proper use of personal protective equipment (PPE), the risks to themselves and their families, and the infection control policies and procedures (e.g., train-the-trainer model, quality assurance monitoring).

Every-day clinical practice has also changed for most of medical and surgical specialties: elective procedures, non-COVID-19 wards and activities, laboratories, and routine areas were everywhere reduced or temporary closed or shifted to COVID-19 activities and areas. Personnel was shifted and converted to COVID-19 areas and activities, which require a large sharing of health personnel. Pooling the hospital manpower from different usual specialties was a common choice; shift and crews were reshaped and recommitted to new tasks and functions (mixed emergency room management, new implementation of ultrasound imaging as lung ultrasound) [86]. This revolution in clinical management of medical emergencies and routine eventually changed the whole clinical activity in most of hospitals [87]; this change, however, should not waste all the progress achieved in the management and prevention of non-COVID-19 disease and mainly CVD.

New pathways and clinical guidelines have been developed and issued in order to match the prevention of COVID-19 and high-quality prevention and management of CVD [45, 64, 88–90].

The invasive management of COVID-19-positive patients should be restricted to type-1 MI [5]. Patients presenting with STEMI or very high-risk NSTEMI and COVID-19 infection should access cath lab with optimal protection for the staff [91, 92]. In case of suspected COVID-19, in STEMI or high-risk NSTEMI patients, such patients should be managed assuming a positive COVID-19 status. The hub and spoke STEMI network should be reshaped: specific COVID-19 hospitals or wards should be set up in large community tertiary hospitals serving as STEMI hubs with intensive care and/or coronary care units and a 24 h/7d catheter laboratory activity; non-COVID-19 patients should be referred to COVID-19-free hospitals or wards (within a hub center).

The current care pathways for chronic elective CV patients have also changed during COVID-19 pandemic for safety reasons. Visits were often changed into telephone and video consultations. Any non-essential diagnostic investigation has been usually deferred [73], while CV emergencies and non-deferrable procedures should be warranted [93]. Telehealth frameworks for HF patients, based on the telemonitoring as proposed by the ESC HF Guidelines, were implemented during pandemic [94, 95]. In Italy, Salzano et al. described the successful implementation of telemedicine service for HF patients, which was provided throughout lockdown with 58% of patients accessing the service [96]. With the transition to a post-pandemic phase, the emergency telemedicine support should be translated into a stable approach [97]. Remote monitoring may detect underlying disease progression and prevent decompensation in NYHA class III patients [98].

An alternative to telemedicine approach has been also described by some centers, and it has been demonstrated that relocating HF services to isolated areas of admission on peripheral specialty units has prevented transmission of COVID-19 in advanced HF patients [99]. Color-coded zones have been also implemented in an HF unit in Milan, Italy, to ensure the isolation of infected, suspected, and COVID-free patients [100].

## Conclusions

Cardiac manifestations of COVID-19 may include cardiac arrhythmias, myocarditis, pericarditis, ACS, HF, cardiogenic shock, and cardiac arrest. All these conditions can occur in absence of pulmonary complications, long after viral clearance and recovery, and be associated with higher mortality rates. Stay-at-home mandates and fear of contagion in hospital have discouraged access to emergency medical services and to ambulatory care, even in patients with severe CVD. In the next future, a change in the phenotype of CV patients is expected, with more advanced HF patients, arrest cardiac, worsening comorbidities, negative ventricular remodeling and reduced EF, more patients with permanent atrial fibrillation. The treatment of such patients and complications may represent the new challenge for cardiologists, presumably requiring a wide reorganization of cardiology departments and cardiovascular care.

## Data Availability

This is a review paper; data availability in not applicable.
